# Preeclampsia Prevention by Timed Birth at Term

**DOI:** 10.1161/HYPERTENSIONAHA.122.20565

**Published:** 2023-04-10

**Authors:** Laura A. Magee, David Wright, Argyro Syngelaki, Peter von Dadelszen, Ranjit Akolekar, Alan Wright, Kypros H. Nicolaides

**Affiliations:** Institute of Women and Children’s Health, School of Life Course and Population Sciences (L.A.M., P.v.D.), King’s College Hospital, London, United Kingdom.; Fetal Medicine Research Institute (A.S., K.H.N.), King’s College Hospital, London, United Kingdom.; Institute of Health Research, University of Exeter, United Kingdom (D.W., A.W.).; Fetal Medicine Unit, Medway Maritime Hospital, Gillingham, United Kingdom (R.A.).; Institute of Medical Sciences, Canterbury Christ Church University, Chatham, United Kingdom (R.A.).

**Keywords:** competing-risks model, induction, number-needed-to-deliver, preeclampsia, risk

## Abstract

Most preeclampsia occurs at term. There are no effective preventative strategies. We aimed to identify the optimal preeclampsia screening and timing of birth strategy for prevention of term preeclampsia.

**Methods:**

This secondary analysis was of data from a prospective nonintervention cohort study of singleton pregnancies delivering at ≥24 weeks, without major anomalies, at 2 United Kingdom maternity hospitals. At routine visits at 11 to 13 weeks’ (57 131 pregnancies screened, 1138 term preeclampsia developed) or 35 to 36 weeks’ gestation (29 035 pregnancies screened, 619 term preeclampsia), with patient-specific preeclampsia risks determined by: United Kingdom National Institute for Health and Care Excellence guidance, and the Fetal Medicine Foundation competing-risks model. For each screening strategy, timing of birth for term preeclampsia prevention was evaluated at gestational time points that were fixed (37, 38, 39, 40 weeks) or dependent on preeclampsia risk by the competing-risks model at 35 to 36 weeks. Main outcomes were proportion of term preeclampsia prevented, and number-needed-to-deliver to prevent one term preeclampsia case.

**Results:**

The proportion of term preeclampsia prevented was the highest, and number-needed-to-deliver lowest, for preeclampsia screening at 35 to 36 (rather than 11–13) weeks. For delivery at 37 weeks, fewer cases of preeclampsia were prevented for National Institute for Health and Care Excellence (28.8%) than the competing-risks model (59.8%), and the number-needed-to-deliver was higher (16.4 versus 6.9, respectively). The risk-stratified approach (at 35–36 weeks) had similar preeclampsia prevention (by 57.2%) and number-needed-to-deliver (8.4), but fewer women would be induced at 37 weeks (1.2% versus 8.8%).

**Conclusions:**

Risk-stratified timing of birth at term may more than halve the risk of term preeclampsia.

**Novelty and Relevance**

**What Is New?**

Risk-stratified timing of birth at term may prevent term preeclampsia, for which no intervention has been proven effective.

**What Is Relevant?**

Term preeclampsia represents at least 75% of all preeclampsia, a leading cause of maternal and perinatal mortality and morbidity, especially in under-resourced settings. Timed birth through labor induction is a widely-available intervention.

**Clinical/Pathophysiological Implications?**

Prevention of preeclampsia has potential to improve adverse pregnancy outcomes associated with term preeclampsia, but a randomized trial is required to confirm that early term birth is not associated with an increase in short-term neonatal (particularly respiratory) morbidity. Also, with successful prevention of term preeclampsia, we will need to find ways of addressing these women’s underlying cardiovascular risk, driven by shared risk factors for cardiovascular disease.

**See editorial, pp 979–980**

Preeclampsia complicates 2% to 4% of pregnancies and is a leading cause of maternal and perinatal mortality and morbidity, globally.^1^ About one quarter of preeclampsia occurs at preterm gestational age, when there is a higher per-pregnancy risk of complications, compared with term disease. However, term preeclampsia is at least 3 times more common, so more than half of maternal, and a substantial proportion of perinatal, adverse outcomes occur in association with term disease.^2^ In the United States, it has been estimated that term preeclampsia is responsible for about one-third of the 6.4 billion dollars (2012) in maternal-infant costs of preeclampsia.^3^

There are effective strategies only for preterm (not term) preeclampsia prevention. Using the Fetal Medicine Foundation competing-risks model at 11 to 13 weeks’ gestation (the strategy associated with the highest detection rate for preterm PE^4^) to identify women at high risk for preterm preeclampsia, treatment with low-dose aspirin decreases preterm preeclampsia by almost two-thirds.^5^ However, as cases of preterm preeclampsia prevented are likely to occur as term preeclampsia,^5^ aspirin has little or no effect on the overall incidence of term preeclampsia. Similarly, using the competing-risks model at 35 to 36 weeks to identify women at high risk (the strategy associated with the highest detection rate for term PE^6^), treatment with pravastatin does not reduce term preeclampsia or associated complications.^7^

Planned delivery at term is an intervention worthy of consideration in women at high-risk for preeclampsia, based on the promising results of the ARRIVE trial; low-risk nulliparous women offered induction at 39 weeks (versus ongoing expectant care) less often developed gestational hypertension or preeclampsia.^7^ For women identified in early pregnancy as being at high risk for preterm preeclampsia and treated with aspirin, ARRIVE has fuelled interest in timed birth at term, at 39 (NCT05056467) or 40 weeks.^8^

In this analysis, we investigated combinations of preeclampsia screening approaches and timed birth strategies to prevent term preeclampsia. For screening, we evaluated clinical risk factor scoring, the competing-risks model at each of 11 to 13 and 35 to 36 weeks, and a risk-stratified approach based on the competing-risks model. For timed birth, we evaluated strategies at fixed points for all high-risk women (at 37, 38, 39, or 40 weeks) and time points dependent on preeclampsia risk.

## Methods

### Data Availability

Data supporting findings of this study are available through collaboration from the Fetal Medicine Foundation (fmf@fetalmedicine.org), upon reasonable request.

### Study Populations

We undertook secondary analyses of data from 2 cohorts of women who attended routine hospital visits at King’s College Hospital, London and Medway Maritime Hospital, Gillingham, United Kingdom, at 11 to 13 weeks (57 131 women, March, 2006 to March, 2017)^9^ or 35 to 36 weeks’ gestation (29 035 women, October, 2016 to September, 2018),^10^ All women gave written informed consent to participate, approved by the NHS Research Ethics Committee. There was no patient involvement in study design, but in a survey of 100 women attending their third trimester fetal ultrasound scan, >90% stated they would be willing to be cared for by risk-stratified timed birth.

Inclusion criteria for this analysis were singleton pregnancies and delivery of a non-malformed liveborn or stillborn at ≥24 weeks. We excluded pregnancies with aneuploidies and major fetal abnormalities, and in the 11 to 13 week cohort, pregnancies ending in miscarriage, termination, or other fetal death before 24 weeks.

The 11 to 13 weeks visit included recording of maternal demographics and medical history^11^; weight and height; mean arterial pressure by validated automated devices and standardised protocol^12^; left and right uterine artery pulsatility index (UtA-PI) by transabdominal colour Doppler ultrasound and calculation of mean UtA-PI^13^; and serum concentration of placental growth factor (PlGF) and PAPP-A (pregnancy-associated plasma protein-A; DELFIA Xpress system, PerkinElmer Life and Analytical Sciences, USA or BRAHMS KRYPTOR analyzer, Thermo Fisher Scientific, Germany).

The 35 to 36 weeks visit included recording of the following: maternal demographics and medical history^11^; weight and height; MAP^12^; and serum PlGF and serum sFlt-1 (soluble fms-like tyrosine kinase-1) by an automated biochemical analyzer (BRAHMS KRYPTOR compact PLUS; Thermo Fisher Scientific, Germany).

Gestational age was determined by measurement of fetal crown-rump length at 11 to 13 weeks’ gestation or the fetal head circumference at 19 to 24 weeks.

Contemporaneous management of hypertension was to initiate antihypertensive therapy at a blood pressure (BP) of 150/100 mmHg.

### Screening

Preeclampsia risk screening strategies were based on either the UK National Institute for Health and Care Excellence (NICE) guidance for screening by clinical risk factors,^14^ or the Fetal Medicine Foundation competing-risks model, applied at 11 to 13 or 35 to 36 weeks’ gestation.

The UK NICE supports identification in early pregnancy of preeclampsia clinical risk factors. Women are considered at high risk of preeclampsia if they have at least 1 high- or 2 moderate risk factors^14^; in essence, each risk factor is treated as a separate screening test. The 5 high-risk factors are the following: hypertensive disease in previous pregnancy, chronic hypertension, diabetes, chronic kidney disease, and autoimmune disease. The 5 moderate-risk factors are the following: first pregnancy, age >40 years, body mass index at first visit of >35 kg/m^2^, interpregnancy interval >10 years, and family history of preeclampsia.^14^ For term preeclampsia, the detection rate has been estimated to be 34% (95% CI, 27–41), for a screen-positive rate of 10%.^15^ High-risk women are offered low-dose aspirin, which prevents >60% of preterm disease, but it does not reduce the incidence of term disease.^5^

The competing-risks model is a multivariable model for prediction of preeclampsia, available for use through an online calculator (https://fetalmedicine.org/research/assess/preeclampsia/). At 11 to 13 weeks, the model includes maternal demographics and medical history, mean arterial pressure, UtA-PI, and PlGF (but PAPP-A can be used if available from aneuploidy screening^9^); neither PlGF nor PAPP-A shows good performance for prediction of term preeclampsia (Table S1). At 35 to 36 weeks, the competing-risks model includes maternal demographics and medical history, mean arterial pressure, PlGF, and sFlt-1; for a screen-positive rate of 10%, 75% of subsequent preeclampsia can be detected.^1^ Alternatively, at 35 to 36 weeks, risk can be divided into 5 strata for term preeclampsia: (≥1 in 2), (1 in 2, to 1 in 5), (1 in 5, 1 in 20), (1 in 20, 1 in 50), and (<1 in 50).^16^

### Preeclampsia Diagnosis

Outcome data were collected from hospital maternity or general medical practitioners’ records. preeclampsia was defined as per the American College of Obstetricians and Gynecologists.^17^ Preeclampsia was defined as chronic or gestational hypertension, and development of at least one of new-onset proteinuria, serum creatinine >97 µmol/L in the absence of underlying renal disease, serum transaminases more than twice normal (≥65 IU/L for our laboratory), platelet count <100 000/µL, headache or visual symptoms, or pulmonary edema.^17^ Chronic hypertension was (systolic BP ≥140 mmHg and/or diastolic BP ≥90 mmHg, at least twice, 4 hours apart), documented before pregnancy or <20 weeks.^18^ Gestational hypertension was defined as new-onset hypertension developing at ≥20 weeks in a previously normotensive woman.^17^

Gestational age at birth was determined for women in the high-risk stratum according to each screening strategy and for women who developed preeclampsia at term.

### Timed Birth

We evaluated 2 timing of birth strategies for prevention of term preeclampsia (Table 1).

**Table 1.**
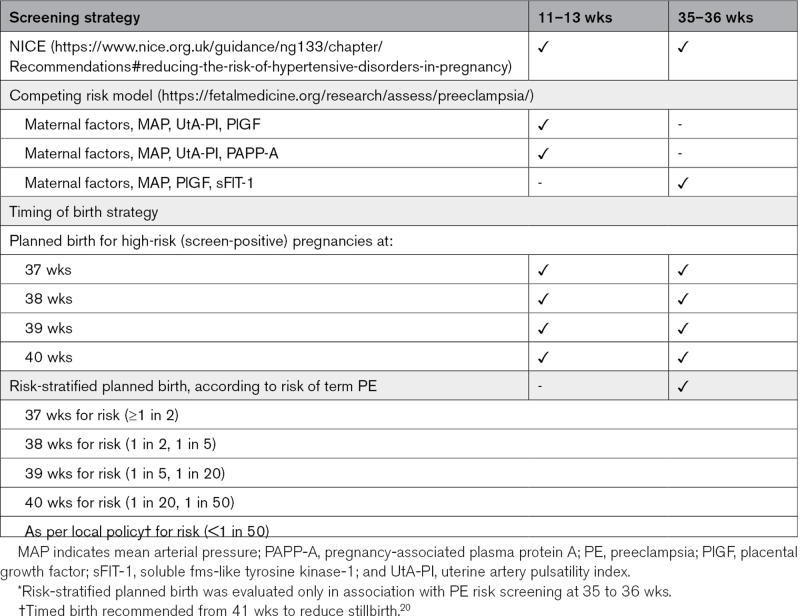
Proposed Screening and Timing of Birth Strategies for Prevention of Term PE*

The first strategy was planned birth at a specific gestational age time point (of 37, 38, 39, or 40 weeks), for all high-risk (screen-positive) women identified at 11 to 13 or 35 to 36 weeks. The time points of 37 and 38 weeks were chosen as the earliest possibilities for timed birth at term, recognizing that they could increase neonatal morbidity, particularly at 37 weeks.^19^ Thirty-nine and 40 weeks were chosen to maximize the opportunity for women to go into spontaneous labor and minimize any potential neonatal morbidity.^20^ We did not evaluate timed birth at 41 weeks, as this is already recommended to reduce the incidence of stillbirth, for the just under 20% of women who are yet to go into spontaneous labor.^20^

The second strategy was personalized, risk-stratified planned birth, according to the risk of preeclampsia determined at 35 to 35 weeks, in 5 strata (Table 1).^16^

### Analysis

Data were summarized descriptively for the total population and by preeclampsia risk status. Median and interquartile range was used for continuous variables and number (percentage) for categorical variables.

For each screening strategy, we determined the detection rate and 95% CI for term preeclampsia.

For each timing of birth strategy, we assumed that timed birth at a given gestational age could prevent half of the term preeclampsia cases that occur within that 1-week epoch, as well as all preeclampsia cases that occur thereafter. Similarly, we assumed that half of births in a given one-week gestational age epoch would be timed, as well as all those thereafter; this was based on a 3-day induction booking window required in clinical care. The number-needed-to-deliver to prevent one case of term preeclampsia was calculated as the number of initiation of births required, divided by the number of term preeclampsia cases prevented.

No power calculation was undertaken.

### Funder’s Role

Funders had no role in design, data collection, analysis, interpretation of results, write-up, or decision to submit.

## Results

Table 2 presents baseline characteristics, preeclampsia screening details, and pregnancy outcomes for pregnancies screened at 11 to 13 weeks (N=57 131) or 35 to 36 weeks (N=29 035), as previously published.^9,10^ Most women screened at 11 to 13 weeks were in their early 30s, and self-identified as being of White race, with 17% Black women, and smaller proportions of South Asian, East Asian, and Mixed-race women. On average, body mass index was at the upper limits of normal. Few women (<3%) had prior chronic hypertension, diabetes, or autoimmune disease. Almost 10% were smokers. A small proportion (3·9%) reported a family history of preeclampsia. Fewer than 5% of conceptions were by assisted means and just over half of women were parous, with 3·0% overall reporting prior preeclampsia. Interpregnancy interval was about 3 years. Women screened at 35 to 36 weeks were similar with regard to demographics and pregnancy characteristics, although average body mass index was higher (in the overweight range), and there were fewer women of Black race, although they still comprised 10% of the screened population.

**Table 2.**
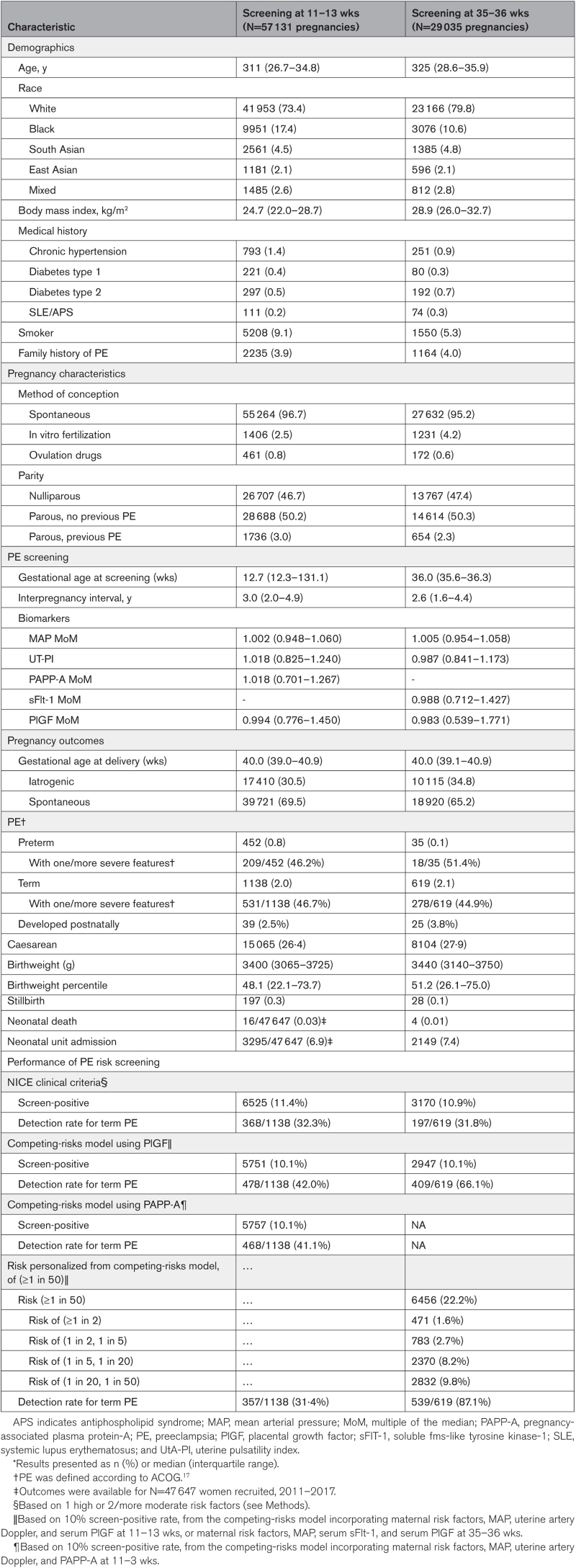
Baseline Characteristics, PE Screening Strategies, and Pregnancy Outcomes*

Pregnancy outcomes were similar in the 2 screening cohorts (Table 2). On average, delivery was at 40 weeks, with about two-thirds following spontaneous onset of labor, and with Cesarean as the mode of birth in one-quarter of women. Preterm preeclampsia occurred more often following 11 to 13 week screening (0.8%) than after 35 to 36 week screening (0.1%), but term preeclampsia occurred with similar frequency (1138, 2.0% and 619, 2.1%, respectively). Average birthweight and birthweight centile were slightly higher in the 35 to 36 week cohort. Stillbirth and neonatal deaths were rare in each cohort, but neonatal unit admission was similar, at about 7%.

The preeclampsia screening strategies at 11 to 13 weeks yielded similar “screen-positive” rates (of ≈10%) and detection rates for term preeclampsia that were lower with NICE (32.3%) than with the competing-risks model using PAPP-A (41.1%) or PlGF (42.0%; Table 2).

At 35 to 36 weeks, the screen-positive and detection rates for term preeclampsia varied by screening strategy: NICE (10.9% and 31.8%, respectively), the competing-risks model (10.1% and 66.1%, respectively), and the competing-risks model using stratified risk (22.2% and 87.1%, respectively; Table 2).

### Preeclampsia Screening and Timed Birth

For various screening and timing of birth strategies, Table 3 presents the proportion of term preeclampsia cases prevented, the planned deliveries required, and the associated number-needed-to-deliver to prevent one case of term preeclampsia. Calculations were based on the gestational age at birth with term preeclampsia and gestational age at birth for all women who screened-positive by a given strategy at 11 to 13 weeks’ (Table S2) and 35 to 36 weeks’ gestation (Table S3).

**Table 3.**
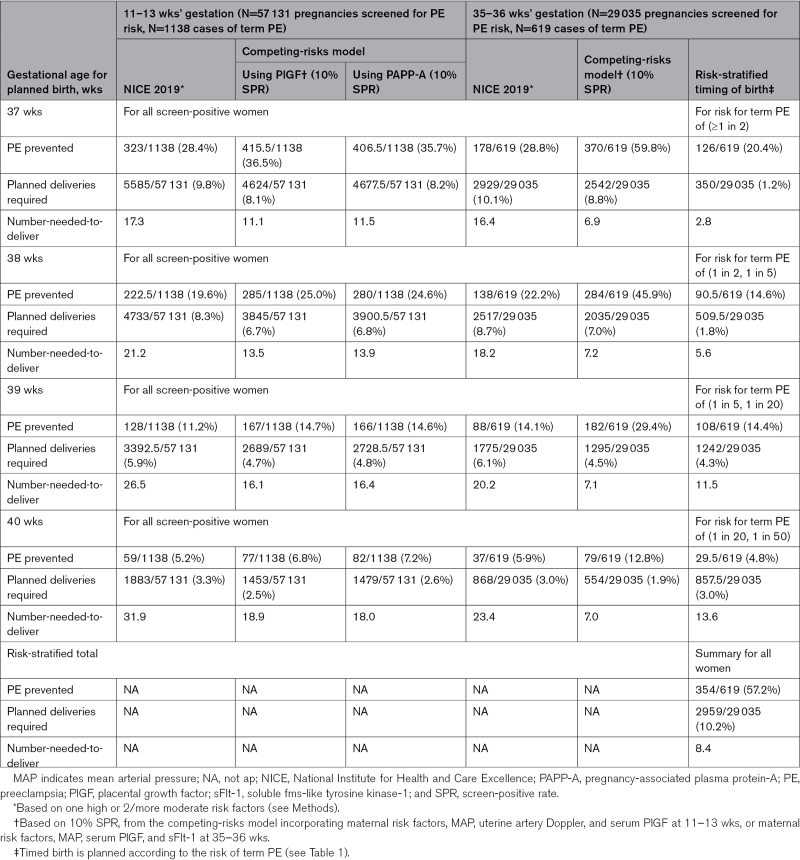
Number-Needed-to-Deliver to Avoid One Case of Term PE, According to Various Screening and Timing of Birth Strategies

Using screening for preeclampsia risk at 11 to 13 weeks’ gestation, and NICE criteria to identify high-risk pregnancies, prevention of term preeclampsia varied from 28.4% at 37 weeks to 5.2% at 40 weeks, planned deliveries required varied from 9.8% to 3.3%, respectively, and number-needed-to-deliver varied from 17.3 to 31.9, respectively (Table 3). The competing-risks model results were similar with use of PAPP-A or PlGF. With use of PlGF, prevention of term preeclampsia varied from 36.5% at 37 weeks to 6.8% at 40 weeks, with the number-needed-to-deliver varying from 11.1 to 18.9, respectively. With use of PAPP-A, cases of term preeclampsia prevented fell from 35.7% at 37 weeks to 7.2%, with the number-needed-to-deliver rising from 11.5 to 18.0, respectively.

For the 35 to 36 week cohort, and NICE criteria, the proportion of term preeclampsia prevented and number-needed-to-deliver values were similar to those for the 11 to 13 week cohort, varying from 28.8% of term preeclampsia prevented (and number-needed-to-deliver of 16.4) to 5.9% of term preeclampsia prevented (for number-needed-to-deliver of 23.4; Table 3). Using the competing-risks model (and a fixed screen-positive rate of 10%), prevention of term preeclampsia varied from 59.8% at 37 weeks to 12.8% at 40 weeks, with the number-needed-to-deliver just under half that with use of NICE criteria (ie, from 6.9 at 37 weeks to 7.0 at 40 weeks). Using a personalized timing of birth strategy based on risks of (≥1 in 50) from the competing-risks model, prevention of term preeclampsia rates and number-needed-to-deliver varied by risk stratum, for an overall prevention of term preeclampsia of 57.2% and number-needed-to-deliver of 8.4 (bottom of Table 3). While the highest proportion of term preeclampsia (59·8%) was prevented by timed birth at 37 weeks for all women identified as being at high risk by the competing-risks model, this was estimated to require birth at 37 weeks for 8.8% of women, rather than the 1.2% with a risk-stratified approach, which still prevented over half of term preeclampsia. Figure presents graphically the proportions of term preeclampsia prevented and planned deliveries required for 35 to 36 week screening (for term preeclampsia risk) and timing of birth strategies presented in Table 3.

**Figure.**
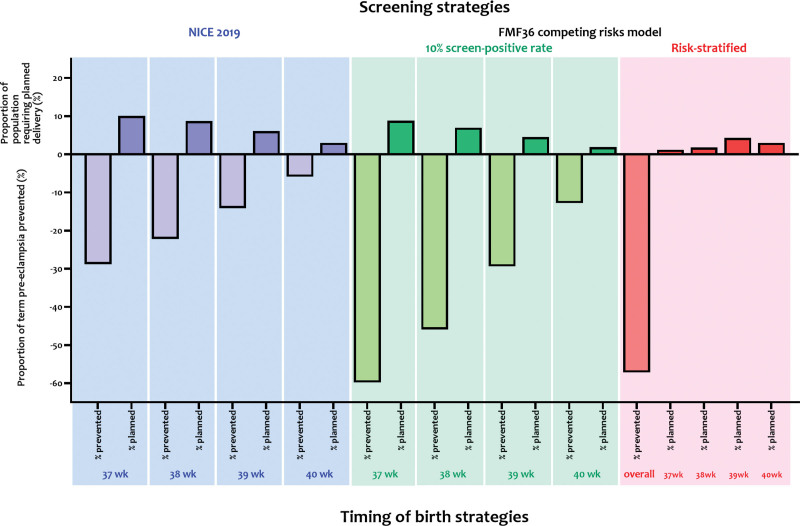
Proportion of term preeclampsia (PE) prevented and planned deliveries required for PE screening and timing of birth strategies.

## Discussion

### Summary of Results

In this prospective screening study of ≈90 000 pregnancies in ethnically diverse South East England, we demonstrated that planned early term birth may reduce the risk of term preeclampsia, with associated numbers-needed-to-deliver that are low and dependent on both preeclampsia screening and timing of birth strategies.

Preeclampsia screening at 11 to 13 (versus 35–36) weeks was associated with higher numbers-needed-to-deliver and lower proportions of preeclampsia prevented for timed birth at fixed time points, from 37 to 40 weeks. Numbers-needed-to-deliver were highest (17.3–31.9) when screening for preeclampsia risk was undertaken by NICE clinical criteria. While numbers-needed-to-deliver were a third to a half lower when preeclampsia screening was done by the competing-risks model, there was little difference between use of PlGF (numbers-needed-to-deliver of 11.1–18.9) or PAPP-A (numbers-needed-to-deliver of 11.5–18.0) and each prevented at most 36% of term preeclampsia. As such, performance of all timed delivery approaches at term to prevent term preeclampsia was inferior when risk for term preeclampsia was determined at 11 to 13 (versus 35–36) weeks.

While preeclampsia screening at 35 to 36 weeks was undertaken on a different cohort of women than those evaluated at 11 to 13 weeks, the numbers-needed-to-deliver and proportion of preeclampsia prevented were similar when NICE criteria (all of which are available in early pregnancy) were used. The lowest number-needed-to-deliver (6.9) was seen with timed delivery at 37 weeks for women at high-risk by the competing-risks model; 59.8% of term preeclampsia was prevented, with 8·8% of the population induced to achieve this. With use of a risk-stratified approach, for screening (competing-risks model) and timed birth, the number-needed-to-deliver for timed birth was slightly higher (8.4) and resulted in a similar proportion of term preeclampsia cases prevented (57.2%) but with far fewer women induced at 37 weeks (1.2%).

### Comparison With Literature

Most preeclampsia occurs at term. In a population-based study of >1 million births in Canada, severe forms of preeclampsia occurred in similar absolute numbers of women at term and preterm gestational ages.^21^

There is currently no effective strategy for prevention of term preeclampsia. For women identified at 11 to 13 weeks as high-risk for preeclampsia by the competing-risks model, aspirin has no effect on term PE^5^; and this approach identifies as high-risk less than half of women who will develop term preeclampsia. Therefore, it is not surprising that the 11 to 13 weeks competing-risks model using PlGF performs similarly and poorly, compared with the model using PAPP-A. For women at high risk of preeclampsia at 35 to 36 weeks by the competing-risks model, pravastatin did not reduce development of term preeclampsia or other adverse outcomes.^7^

Timed birth at term represents a potential strategy for preeclampsia prevention, based on the effectiveness of this intervention for management of preeclampsia (and gestational hypertension) at term.^22^ For women with chronic or gestational hypertension at term, observational data^23,24^ and small subgroups within trials^22,25^ suggest that planned early term delivery may benefit the mother; a trial is ongoing (REGISTRATION: URL: https://www.clinicaltrials.gov; Unique identifier: ISRCTN77258279).

Timed birth at term has been shown to decrease term preeclampsia in nulliparous women (who are at increased risk of preeclampsia). In a systematic review of ARRIVE (6106 women)^26^ and 15 other trials (2690 women) of nulliparous women at term, labor induction (usually at 38–40 weeks), versus expectant care, reduced development of gestational hypertension or preeclampsia, with shorter first stages of labor and fewer babies with meconium-stained amniotic fluid, but without an impact on other perinatal outcomes or cesareans; however, timed birth was associated with more frequent use of epidural analgesia, longer maternal hospitalization, and lower birth weight.^27^

There are no published trials of planned early term delivery in women at high risk of preeclampsia, based on NICE clinical criteria or the competing-risks model.

There is no compelling evidence that maternal risk is increased by induction, and randomized trials have demonstrated that induction decreases cesareans.^28^ Although observational data on elective deliveries before 39 weeks have documented excess neonatal mortality/morbidity^19^ and possibly, special educational needs above the baseline of ~5%,^29^ trials of timed birth at term have been reassuring. In a Cochrane review (34 trials, >21 000 women), labor induction from 37 weeks (versus expectant care) was associated with fewer perinatal deaths (69% reduction), stillbirths (70% reduction), babies requiring intensive care (12% reduction), and cesareans (10% reduction)^30^; no data were reported on child neurodevelopment. There were no differences between gestational age subgroups, or by parity or cervical status. The number-needed-to-induce to prevent one perinatal death was 544 (95% CI, 441–1042).

### Strengths and Limitations

A strength of our study is inclusion of a large population of women, carefully phenotyped and screened for preeclampsia in a standardized fashion. Clinicians caring for women in the cohort were not aware of their preeclampsia risk status by the competing-risks model, which did not affect their gestational age at delivery.

Limitations include that clinicians were aware of clinical risk factors for preeclampsia, as is ethical. We assumed that at a given gestational age, induction during a 1-week epoch could both prevent half of the term preeclampsia cases and require induction for half of the number of deliveries that would have otherwise occurred during that week; we think this is reasonable given that labor induction are booked over the first few days of a gestational week, and it takes about 2 days for birth, and women in higher- (versus lower-) risk strata for preeclampsia go into spontaneous labor more often^16^; however, we recognize that our modeling does not take into account that preeclampsia could still develop postpartum. Finally, observational studies such as ours are potentially confounded by indication for delivery, so it is not possible to estimate with any certainty the impact of different gestations at delivery on perinatal outcomes. This would require a randomized trial.

### Conclusions

Risk-stratified timing of birth at term is likely to more than halve the risk of term preeclampsia, with fewer than 10 inductions required per case avoided. A randomized trial is needed to evaluate the effectiveness and perinatal safety of this intervention, to ensure that a policy shift represents an intervention that confers benefits and is cost-effective.

### Perspectives

Most preeclampsia occurs at term, when the majority of maternal and a substantial proportion of associated perinatal short-term adverse outcomes occur; there is also increased cardiovascular risk long-term, for mothers and babies. As such, term preeclampsia is an outcome worthy of avoidance, rather than one to which we should only react when it develops. Risk-stratified timing of birth could prevent more than half of term preeclampsia, based on consideration of known maternal characteristics, BP, and angiogenic markers (sFlt-1 and PlGF). This intervention may be particularly useful in under-resourced settings, where the capacity to care for ill mothers with preeclampsia is particularly challenging, where the vast majority of women with preeclampsia die, and where timed birth can be offered easily and inexpensively. However, whether any maternal and fetal benefits would outweigh any increase in neonatal morbidity must be established by randomized trials.

## Article Information

### Author Contributions

All authors conceptualized and designed the study. L.A. Magee wrote the first article draft. A. Syngelaki and R. Akolekar were involved in sample collection. A. Syngelaki accessed and verified the data. D. Wright and A. Wright provided statistical support. All authors had full access to the data, revised and contributed to the intellectual content of the article, and approved its submission.

### Acknowledgments

The study was conducted according to the guidelines of the Declaration of Helsinki and approved by the NHS Research Ethics Committee (reference: 02-03-033, 11/March/2003).

### Sources of Funding

This study was supported by grants from the Fetal Medicine Foundation (UK Charity 1037116). Reagents and relevant equipment were provided free-of-charge for serum placental growth factor (PerkinElmer Life and Analytical Sciences, USA; Roche Diagnostics, Germany; Thermo Fisher Scientific, Germany); and sFlt-1 (Thermo Fisher Scientific). These bodies had no involvement in: study design; data collection, analysis; interpretation; results write-up; or the decision to submit for publication.

### Disclosures

None.

### Supplementary Material



## References

[1] Magee LA Nicolaides KH von Dadelszen P. Preeclampsia. N Engl J Med. 2022;386:1817–1832. doi: 10.1056/NEJMra2109523 35544388 10.1056/NEJMra2109523

[2] von Dadelszen P Syngelaki A Akolekar R Magee LA Nicolaides KH. Preterm and term pre-eclampsia: relative burdens of maternal and perinatal complications [published online December 23, 2022]. BJOG. doi: 10.1111/1471-0528.17370. https://obgyn.onlinelibrary.wiley.com/doi/10.1111/1471-0528.17370 10.1111/1471-0528.17370 36562190

[3] Stevens W Shih T Incerti D Ton TGN Lee HC Peneva D Macones GA Sibai BM Jena AB. Short-term costs of preeclampsia to the United States health care system. Am J Obstet Gynecol. 2017;217:237–248.e16. doi: 10.1016/j.ajog.2017.04.032 28708975 10.1016/j.ajog.2017.04.032

[4] Wright D Wright A Nicolaides KH. The competing risk approach for prediction of preeclampsia. Am J Obstet Gynecol. 2020;223:12–23.e7. doi: 10.1016/j.ajog.2019.11.1247 31733203 10.1016/j.ajog.2019.11.1247

[5] Rolnik DL Wright D Poon LC O’Gorman N Syngelaki A de Paco Matallana C Akolekar R Cicero S Janga D Singh M. Aspirin versus placebo in pregnancies at high risk for preterm preeclampsia. N Engl J Med. 2017;377:613–622. doi: 10.1056/NEJMoa1704559 28657417 10.1056/NEJMoa1704559

[6] Panaitescu A Ciobanu A Syngelaki A Wright A Wright D Nicolaides KH. Screening for pre-eclampsia at 35-37 weeks’ gestation. Ultrasound Obstet Gynecol. 2018;52:501–506. doi: 10.1002/uog.19111 29896778 10.1002/uog.19111

[7] Dobert M Varouxaki AN Mu AC Syngelaki A Ciobanu A Akolekar R De Paco Matallana C Cicero S Greco E Singh M. Pravastatin versus placebo in pregnancies at high risk of term preeclampsia. Circulation. 2021;144:670–679. doi: 10.1161/CIRCULATIONAHA.121.053963 34162218 10.1161/CIRCULATIONAHA.121.053963

[8] Guy GP Leslie K Diaz Gomez D Forenc K Buck E Khalil A Thilaganathan B. Implementation of routine first trimester combined screening for pre-eclampsia: a clinical effectiveness study. BJOG. 2021;128:149–156. doi: 10.1111/1471-0528.16361 32613730 10.1111/1471-0528.16361

[9] Mazer Zumaeta A Wright A Syngelaki A Maritsa VA Da Silva AB Nicolaides KH. Screening for pre-eclampsia at 11-13 weeks’ gestation: use of pregnancy-associated plasma protein-A, placental growth factor or both. Ultrasound Obstet Gynecol. 2020;56:400–407. doi: 10.1002/uog.22093 32441401 10.1002/uog.22093

[10] Lai L Syngelaki A Nicolaides KH von Dadelszen P Magee LA. Impact of new definitions of preeclampsia at term on identification of adverse maternal and perinatal outcomes. Am J Obstet Gynecol. 2021;224:518.e1–518.e11. doi: 10.1016/j.ajog.2020.11.004 10.1016/j.ajog.2020.11.004 33166504

[11] Wright D Syngelaki A Akolekar R Poon LC Nicolaides KH. Competing risks model in screening for preeclampsia by maternal characteristics and medical history. Am J Obstet Gynecol. 2015;213:62.e162 e1–62.e62.e10. doi: 10.1016/j.ajog.2015.02.018 10.1016/j.ajog.2015.02.018 25724400

[12] Poon LC Zymeri NA Zamprakou A Syngelaki A Nicolaides KH. Protocol for measurement of mean arterial pressure at 11-13 weeks’ gestation. Fetal Diagn Ther. 2012;31:42–48. doi: 10.1159/000335366 22248988 10.1159/000335366

[13] Plasencia W Maiz N Bonino S Kaihura C Nicolaides KH. Uterine artery Doppler at 11 + 0 to 13 + 6 weeks in the prediction of pre-eclampsia. Ultrasound Obstet Gynecol. 2007;30:742–749. doi: 10.1002/uog.5157 17899573 10.1002/uog.5157

[14] NICE. Hypertension in pregnancy: diagnosis and management. 2019. https://www.nice.org.uk/guidance/ng133.

[15] O’Gorman N Wright D Poon LC Rolnik DL Syngelaki A Wright A Akolekar R Cicero S Janga D Jani J. Accuracy of competing-risks model in screening for pre-eclampsia by maternal factors and biomarkers at 11-13 weeks’ gestation. Ultrasound Obstet Gynecol. 2017;49:751–755. doi: 10.1002/uog.17399 28067011 10.1002/uog.17399

[16] von Dadelszen P Syngelaki A Wright A Akolekar R Magee LA Wright D Nicolaides KH. The implications of the Fetal Medicine Foundation 35-36 week preeclampsia prediction competing risk model on timing of birth. [published online October 4, 2022]. Am J Obstet Gynecol. doi: 10.1016/j.ajog.2022.09.047. https://www.ajog.org/article/S0002-9378(22)00804-3/fulltext 10.1016/j.ajog.2022.09.047 36206987

[17] ACOG Practice Bulletin No. 202 summary: gestational hypertension and preeclampsia. Obstet Gynecol. 2019;133:211–214. doi: 10.1097/AOG.0000000000003019 10.1097/AOG.0000000000003019 30575668

[18] ACOG Practice Bulletin No. 203: chronic hypertension in pregnancy. Obstet Gynecol. 2019;133:e26–e50. doi: 10.1097/AOG.0000000000003020 30575676 10.1097/AOG.0000000000003020

[19] Zhang X Kramer MS. Variations in mortality and morbidity by gestational age among infants born at term. J Pediatr. 2009;154:358–62, 362.e1. doi: 10.1016/j.jpeds.2008.09.013 18950794 10.1016/j.jpeds.2008.09.013

[20] Inducing labour. NICE guideline (ng207). 2021. https://wwwniceorguk/guidance/ng207.

[21] Lisonkova S Bone JN Muraca GM Razaz N Wang LQ Sabr Y Boutin A Mayer C Joseph KS. Incidence and risk factors for severe preeclampsia, hemolysis, elevated liver enzymes, and low platelet count syndrome, and eclampsia at preterm and term gestation: a population-based study. Am J Obstet Gynecol. 2021;225:538.e1–538.e19. doi: 10.1016/j.ajog.2021.04.261 10.1016/j.ajog.2021.04.261 33974902

[22] Koopmans CM Bijlenga D Groen H Vijgen SM Aarnoudse JG Bekedam DJ van den Berg PP de Boer K Burggraaff JM Bloemenkamp KW; HYPITAT study group. Induction of labour versus expectant monitoring for gestational hypertension or mild pre-eclampsia after 36 weeks’ gestation (HYPITAT): a multicentre, open-label randomised controlled trial. Lancet. 2009;374:979–988. doi: 10.1016/S0140-6736(09)60736-4 19656558 10.1016/S0140-6736(09)60736-4

[23] Hutcheon JA Lisonkova S Magee LA Von Dadelszen P Woo HL Liu S Joseph KS. Optimal timing of delivery in pregnancies with pre-existing hypertension. BJOG. 2011;118:49–54. doi: 10.1111/j.1471-0528.2010.02754.x 21054760 10.1111/j.1471-0528.2010.02754.x

[24] Cruz MO Gao W Hibbard JU. What is the optimal time for delivery in women with gestational hypertension?. Am J Obstet Gynecol. 2012;207:214.e1–214.e6. doi: 10.1016/j.ajog.2012.06.009 10.1016/j.ajog.2012.06.009 22831812

[25] Hamed HO Alsheeha MA Abu-Elhasan AM Abd Elmoniem AE Kamal MM. Pregnancy outcomes of expectant management of stable mild to moderate chronic hypertension as compared with planned delivery. Int J Gynaecol Obstet. 2014;127:15–20. doi: 10.1016/j.ijgo.2014.04.010 24957533 10.1016/j.ijgo.2014.04.010

[26] Grobman WA Rice MM Reddy UM Tita ATN Silver RM Mallett G Hill K Thom EA El-Sayed YY Perez-Delboy A; Eunice Kennedy Shriver National Institute of Child Health and Human Development Maternal–Fetal Medicine Units Network. Labor Induction versus expectant management in low-risk nulliparous women. N Engl J Med. 2018;379:513–523. doi: 10.1056/NEJMoa1800566 30089070 10.1056/NEJMoa1800566 PMC6186292

[27] Dong S Bapoo S Shukla M Abbasi N Horn D D’Souza R. Induction of labour in low-risk pregnancies before 40 weeks of gestation: a systematic review and meta-analysis of randomized trials. Best Pract Res Clin Obstet Gynaecol. 2022;79:107–125. doi: 10.1016/j.bpobgyn.2021.12.007 35086752 10.1016/j.bpobgyn.2021.12.007

[28] Mishanina E Rogozinska E Thatthi T Uddin-Khan R Khan KS Meads C. Use of labour induction and risk of cesarean delivery: a systematic review and meta-analysis. CMAJ. 2014;186:665–673. doi: 10.1503/cmaj.130925 24778358 10.1503/cmaj.130925 PMC4049989

[29] MacKay DF Smith GC Dobbie R Pell JP. Gestational age at delivery and special educational need: retrospective cohort study of 407,503 schoolchildren. PLoS Med. 2010;7:e1000289. doi: 10.1371/journal.pmed.1000289 20543995 10.1371/journal.pmed.1000289 PMC2882432

[30] Middleton P Shepherd E Morris J Crowther CA Gomersall JC. Induction of labour at or beyond 37 weeks’ gestation. Cochrane Database Syst Rev. 2020;7:CD004945. doi: 10.1002/14651858.CD004945.pub5 32666584 10.1002/14651858.CD004945.pub5 PMC7389871

