# Supplementary material for: Preeclampsia Prevention by Timed Birth at Term
Source: Hypertension. 2023 Apr 10;80(5):969–78. doi: 10.1161/HYPERTENSIONAHA.122.20565 (PMC10112937; doi:10.1161/HYPERTENSIONAHA.122.20565)
Supplement: Supplementary file 1 [file hyp-80-0969-s001.doc]

**ONLINE SUPPLEMENT**

**PRE-ECLAMPSIA PREVENTION BY TIMED BIRTH AT TERM**

Laura A MAGEE^1^, MD

David WRIGHT^2^, PhD

Argyro SYNGELAKI^3^, PhD

Peter VON DADELSZEN^1^, DPhil

Ranjit AKOLEKAR^4,5^, MD

Alan WRIGHT^2^, PhD

Kypros H NICOLAIDES^3^, MD

1. Institute of Women and Children’s Health, School of Life Course and Population Sciences, King’s College London, London, UK
2. Institute of Health Research, University of Exeter, Exeter, United Kingdom
3. Fetal Medicine Research Institute, King’s College Hospital, London, United Kingdom
4. Fetal Medicine Unit, Medway Maritime Hospital, Gillingham, United Kingdom
5. Institute of Medical Sciences, Canterbury Christ Church University, Chatham, United Kingdom

**Corresponding author**

Professor Laura A. Magee

Addison House

Guy’s Campus, Great Maze Pond

London, SE1 1UL

Email: [Laura.A.Magee@kcl.ac.uk](mailto:Laura.A.Magee@kcl.ac.uk)

**Running title:** Pre-eclampsia by timed birth at term

**SUPPLEMENTAL TABLES**

| **Table** | **Title** | **Page number** |
| --- | --- | --- |
| S1 | Screening for PE at 11-13 weeks’ gestation and detection rate for term PE | 3 |
| S2 | Development of term PE (N=1,138) and the associated gestational age at birth, for 57,131 pregnancies screened for PE risk at 11-13 weeks’ gestation | 4 |
| S3 | Development of term PE (N=619) and the associated gestational age birth, for 29,035 pregnancies screened for PE risk at 35-36 weeks’ gestation (619 cases of term PE) | 5 |

**Table S1**: Screening for PE at 11-13 weeks’ gestation and detection rate for term PE (at a 10% screen-positive rate for preterm PE)

|  | **Term PE** | |
| --- | --- | --- |
| **PE screening at 11-13 weeks by the competing-risks model** | **N=1,138** | |
| **Competing risks model** | **Events** | **Detection rate** |
| History | 377 | 33∙1 (30∙4-35∙9) |
| History + MAP | 448 | 39.2 (36∙5-42∙3) |
| History + UtA-PI | 412 | 36∙2 (33∙4-39∙1) |
| History + PlGF | 413 | 36.3 (33∙5-39∙2) |
| History + PAPP-A | 392 | 34.4 (31∙7-37∙3) |
| History + MAP + UtA-PI | 466 | 40.9 (38∙1-43∙9) |
| History + MAP + UtA-PI + PlGF | 475 | 41.7 (38∙9-44∙7) |
| History + MAP + UtA-PI + PAPP-A | 468 | 41.1 (38∙2-44∙0) |

MAP = mean arterial blood pressure; PAPP-A = pregnancy associated plasma protein-A; PE = pre-eclampsia; PlGF = placental growth factor; UtA-PI = uterine artery pulsatility index.

**Table S2**: Development of term PE (N=1,138) and the associated gestational age at birth, for 57,131 pregnancies screened for PE risk at 11-13 weeks’ gestation

| **Screening method** | Gestational age at birth (weeks) | N (%) pregnancies with term PE at delivery  (N=368/1,138 identified) | N (%) pregnancies with delivery at term  (N=5,854/6,525) |
| --- | --- | --- | --- |
| NICE 2019 high-risk (N=6,525, 11.4%) | 37 | 90 | 538 |
|  | 38 | 111 | 1166 |
|  | 39 | 78 | 1515 |
|  | 40 | 60 | 1504 |
|  | 41 | 27 | 960 |
|  | 42 | 2 | 171 |
| Competing risks model using PlGF (10% screen-positive rate) (N=5,751, 10.1%) | Gestational age at birth (weeks) | At delivery with term PE (N=478/1,138 identified) | At delivery at term (weeks) (N=4,882/5,751) |
|  | 37 | 125 | 516 |
|  | 38 | 136 | 1042 |
|  | 39 | 101 | 1270 |
|  | 40 | 78 | 1203 |
|  | 41 | 34 | 769 |
|  | 42 | 4 | 82 |
| Competing risks model using PAPP-A (10% screen-positive rate) (N=5,757, 10.1%) | Gestational age at birth (weeks) | At delivery with term PE (N=468/1,138 identified) | At delivery at term (weeks) (N=4,932/5,757) |
|  | 37 | 123 | 509 |
|  | 38 | 130 | 1045 |
|  | 39 | 98 | 1299 |
|  | 40 | 70 | 1200 |
|  | 41 | 42 | 781 |
|  | 42 | 5 | 98 |

NICE = National Institute for Health and Care Excellence; PAPP-A = pregnancy associated plasma protein-A; PE (pre-eclampsia); PlGF = placental growth factor

**Table S3:** Development of term PE (N=619) and the associated gestational age at birth, for 29,035 pregnancies screened for PE risk at 35-36 weeks’ gestation

| **Screening method** | |  | | |
| --- | --- | --- | --- | --- |
| NICE 2019  (N=3,170, 10.9%) | | Gestational age (weeks) | N (%) pregnancies with term PE at delivery (N=197/619 identified) | N (%) pregnancies with delivery at term (N=3,082/3,170) |
|  |  | 37 | 39 | 307 |
|  |  | 38 | 41 | 516 |
|  |  | 39 | 59 | 969 |
|  |  | 40 | 43 | 845 |
|  |  | 41 | 15 | 384 |
|  |  | 42 | 0 | 61 |
| Competing risks model (10% screen-positive rate) (N=2,947, 10.1%) | | Gestational age (weeks) | N (%) pregnancies with term PE at delivery (N=409/619 identified) | N (%) pregnancies with delivery at term (N=2,748/2,947) |
|  |  | 37 | 79 | 413 |
|  |  | 38 | 92 | 600 |
|  |  | 39 | 113 | 880 |
|  |  | 40 | 92 | 603 |
|  |  | 41 | 32 | 234 |
|  |  | 42 | 1 | 18 |
| Competing risks model (risk stratified) (N=28,512) |  | Gestational age (weeks) | N (%) pregnancies with term PE at delivery (N=619/619 identified) | N (%) pregnancies with delivery at term (N=28,512/28,512) |
|  | {≥1 in 2}  (N=471) |  | (N=153/619 identified) | (N=416) |
|  |  | 37 | 54 | 132 |
|  |  | 38 | 43 | 103 |
|  |  | 39 | 34 | 116 |
|  |  | 40 | 20 | 55 |
|  |  | 41 | 2 | 8 |
|  |  | 42 | 0 | 2 |
|  | (1 in 2, 1 in 5) (N=783) |  | (N=118/619 identified) | (N=716) |
|  |  | 37 | 15 | 123 |
|  |  | 38 | 25 | 167 |
|  |  | 39 | 38 | 253 |
|  |  | 40 | 31 | 131 |
|  |  | 41 | 9 | 39 |
|  |  | 42 | 0 | 3 |
|  | (1 in 5, 1 in 20) (N=2,370) |  | (N=179/619 identified) | (N=2,279) |
|  |  | 37 | 14 | 228 |
|  |  | 38 | 31 | 448 |
|  |  | 39 | 52 | 722 |
|  |  | 40 | 55 | 593 |
|  |  | 41 | 26 | 267 |
|  |  | 42 | 1 | 21 |
|  | (1 in 20, 1 in 50) (N=2,832) |  | (N=89/619 identified) | (N=2,759) |
|  |  | 37 | 9 | 196 |
|  |  | 38 | 17 | 444 |
|  |  | 39 | 19 | 877 |
|  |  | 40 | 29 | 769 |
|  |  | 41 | 11 | 423 |
|  |  | 42 | 4 | 50 |
|  | (<1 in 50) (N=22,579) |  | (N=80/619 identified) | (N=22,342) |
|  |  | 37 | 2 | 915 |
|  |  | 38 | 9 | 2,270 |
|  |  | 39 | 16 | 6,680 |
|  |  | 40 | 32 | 6,918 |
|  |  | 41 | 19 | 5,006 |
|  |  | 42 | 2 | 553 |

NICE = National Institute for Health and Care Excellence; PAPP-A = pregnancy associated plasma protein-A; PE = pre-eclampsia; PlGF = placental growth factor
